# Ruminant nutrition symposium: novel microbial solutions to optimize production efficiency in beef and dairy systems

**DOI:** 10.1093/jas/skaf165

**Published:** 2025-05-15

**Authors:** Bruno I Cappellozza, Reinaldo F Cooke, Rafael C Amaral, Alex Bach, Terry E Engle, Luiz F Ferraretto, Rodrigo S Marques, Alexandra C Miller, Philipe Moriel, João M B Vendramini

**Affiliations:** Novonesis, 2800 Lyngby, Denmark; Department of Animal Science, Texas A&M University, College Station, TX 77843; Novonesis, 2800 Lyngby, Denmark; Department of Animal Science, University of Lleida, E-25198 Lleida, Spain; Institució de Recerca i Estudis Avançats (ICREA), E-08009 Barcelona, Spain; Department of Animal Science, Colorado State University, Fort Collins, CO 80524; Department of Animal and Dairy Sciences, University of Wisconsin, Madison, WI 53706; School of Animal Sciences, Virginia Polytechnic Institute and State University, Blacksburg, VA 24061; Department of Animal Science, Colorado State University, Fort Collins, CO 80524; IFAS | Range Cattle Research and Education Center, University of Florida, Ona, FL 33865; Texas A&M Agrilife Research & Extension Center, Stephenville, TX 76401

**Keywords:** *Bacillus* spp, beef cattle, dairy cattle, microbial solutions, silage

## Abstract

The livestock industry is in search of sustainable technologies that enhance the profitability and sustainability of food animal production. Among these technologies, microbial solutions include living bacteria that may support soil, plant, and animal health. Bacterial inoculants have been the most used additives to modulate whole-plant corn silage fermentation, including homo- and heterofermentative bacteria. Bacterial inoculants can improve fermentation characteristics of grass silage, whereas the wide variation in chemical composition and management practices across cool- and warm-season grasses makes the outcome of inoculation less predictable. Tailored recommendations for bacterial inoculant species and strains, based on species and management practices, are necessary to increase the predictability of silage inoculation, including whole-plant corn and grass silage. Microbial solutions are also used as feed additives for beef and dairy cattle, including *Bacillus*-based direct-fed microbials (**DFM**). Recent advancements in *Bacillus*-based DFM supplementation have shown promise for enhancing cow-calf productivity through improved gut health, immune function, and nutrient metabolism. Incorporating *Bacillus-*based DFM into stocker and feedlot receiving programs can improve feed intake and digestibility, health, and productivity of high-risk cattle. *Bacillus-*based DFM have also been used in feedlot diets to address public concerns with antimicrobial resistance but also to improve cattle performance and reduce the incidence of diseases. Multispecies bacterial-based DFM are promising alternatives to counteract the potentially adverse effects of *Salmonella*, as these DFM supported the integrity of the intestinal epithelial cells under in vitro settings. In dairy cattle, most DFM-related research in mature cows has targeted improvements in rumen fermentation and enterocyte health. In general, improvements in milk production due to supplementation of bacteria-based DFM seem to be more consistent than those obtained with yeast-based DFM. Collectively, microbial solutions have the potential to optimize silage quality, enhance feed intake and digestibility by ruminants, and support cattle health and performance while fostering sustainable practices in beef and dairy production systems.

## Teaser

A Ruminant Nutrition Symposium was held during the American Society of Animal Science 2024 Annual Meeting that focused on “Novel microbial solutions to optimize production efficiency in beef and dairy systems.” This manuscript reviews the information presented during the symposium, including how bacteria-based microbial solutions increase the quality and stability of ensiled forages and how supplementing bacteria-based microbial solutions supports the health and performance of beef and dairy cattle.

## Introduction

The livestock industry is constantly searching for sustainable technologies that promote soil and plant stability, conserve feed quality, and improve animal health and productivity. These efforts aim to reduce the utilization of chemical fertilizers in the soil and antibiotics in the diets of the animals, thus reducing the spread of antimicrobial resistance from animal systems to the human food supply (Singh et al., 2024). Among these technologies, increasing interest and attention has been given to the potential that microbial solutions might have in optimizing the productivity of soil, plants, conserved feed, and animals. For this reason, a Ruminant Nutrition Symposium was held during the American Society of Animal Science 2024 Annual Meeting focused on “Novel microbial solutions to optimize production efficiency in beef and dairy systems.” This manuscript reviews the research-based information that was presented during the symposium. The major themes of the symposium focused on modes of action by which bacteria-based microbial solutions increase the quality and stability of ensiled forages and how supplementing bacteria-based microbial solutions promotes the health and performance of beef and dairy cattle herds.

Before describing the mechanisms by which microbial solutions may benefit agriculture and livestock production, it is important to define the “microbial solutions” terminology. In the soil/plant interaction, these include naturally derived substances or living microorganisms that are applied to plants, seeds, or soil to enhance plant growth, improve nutrient availability, control pests and diseases, or restore and maintain soil health (Glare et al., 2012; Malusá and Vassilev, 2014; du Jardin, 2015). Microbial solutions function by leveraging biological processes such as nutrient cycling, microbial symbiosis, and plant–microbe interactions (Malusá and Vassilev, 2014). For feed conservation such as silages and haylages, microbial solutions are used to improve the quality and stability of the ensiled material while limiting the development and growth of undesired microorganisms (Weinberg and Muck, 1996; Schmidt and Kung, 2010; Comino et al., 2014). From an animal standpoint, microbial solutions comprise of live microorganisms that may confer a health benefit to the host when added to the diet in the correct dosages (FAO/WHO, 2001).

## Microbial Solutions in Forage Preservation

Live microbial culture solutions are often classified as homofermentative or heterofermentative bacterial inoculants when used for forage preservation (Kung et al., 2003). Both types of inoculants often show complimentary and additive effects to promote silage quality.

### Homofermentative bacterial inoculants

These bacterial inoculants are generally used to improve the fermentation profile of the ensiled material to control the growth and development of yeast and molds (Weinberg and Muck, 1996), and to reduce the losses of nutrients during the early phases of the ensiling and fermentation processes (Muck, 2013). Examples of this category of bacterial inoculants include *Lactiplantibacillus plantarum* and *Lactococcus lactis*, which promote the acidification of the ensiled material through the production of lactic acid and a rapid reduction in pH to help ensure silage stability (Kung et al., 2003; Hindrichsen et al., 2012). During the ensiling process, the faster pH is reduced, the earlier the undesired side effects of the fermentation process may be halted, contributing to the improved nutritional profile of the silage (Kung et al., 2003).

### Heterofermentative bacterial inoculants

These bacterial inoculants also produce acetate during their fermentation process that provides protection against some fungi growth and enhances the aerobic stability of silages, including whole-plant corn silage, high-moisture corn silage, and haylages (Kung and Ranjit, 2001; Tabacco et al., 2011; Comino et al., 2014). The acetate produced by heterofermentative bacteria can also limit yeast-induced ethanolic fermentation by inhibiting fungal growth in forages with high sugar concentrations. An important representative of this class of silage inoculants includes the gram-positive *Lentilactobacillus* (formerly known as *Lactobacillus*) *buchneri*.

### Combination of homo- and heterofermentative bacteria

Considering the different modes of action in the fermentation process, the combination of homofermentative and heterofermentative bacteria has gained interest among the scientific community. Saylor et al. (2020) reported longer aerobic stability, greater proportion of soluble crude protein (**CP**), and greater in situ ruminal starch degradation when *L. lactis* and *L. buchneri* were added to high-moisture corn silage. In whole-plant corn silage, *L. lactis* and *L. buchneri* inoculation increased aerobic stability by 150 h, which can be also attributed to the oxygen-scavenging ability of *L. lactis* (Kok et al., 2024). These authors also reported improved apparent total tract dry matter (**DM**), CP, neutral detergent fiber (**NDF**), and starch digestibility when whole-plant corn silage inoculated with *L. lactis* and *L. buchneri* was offered to dairy cows (Kok et al., 2024).

Delaying the final ensiling process for 24 h after harvesting, thus exposing aerobic exposure of the fresh forage, increased nutrient losses and reduced aerobic stability of whole-plant corn silage (Jatkauskas et al., 2024). Addition of *L. lactis* and *L. buchneri* increased silage aerobic stability, reduced ammonia-N (**NH**_**3**_**-N**) concentrations, pH, yeast, and mold counts, and decreased the highest temperature achieved after aerobic exposure (Jatkauskas et al., 2024). The oxygen-scavenging ability of *L. lactis* offers significant benefits in farm-scale systems, particularly during the critical transition of the ensiling process from an oxygen-rich to an oxygen-free environment (Muck et al., 2018). Inclusion of *L. lactis* effectively reduced the presence of aerobic microorganisms when silage closure was delayed, which further enhanced aerobic stability of the silage when combined with the acetic acid production efficiency of *L. buchneri* (Jatkauskas et al., 2024). Hence, the oxygen scavenging by *L. lactis* not only suppresses aerobic microorganisms and accelerates fermentation during ensiling but also improves aerobic stability during the feed-out phase when combined with *L. buchneri* in silages re-exposed to oxygen (Jatkauskas et al., 2024).

## Modulating Grain-Based Silage Fermentation with Bacterial Inoculants

Forages are widely fed to ruminants as silage because ensiling decreases nutrient losses from harvest through storage, facilitates feeding, and makes diet mixing more efficient. However, DM and nutrient losses still occur throughout the production, fermentation, and feeding of the silage. Borreani et al. (2018) underscored multiple factors affecting DM losses during anaerobic fermentation and suggested that ~13% of DM will be lost despite appropriate silage management. Although some losses are unavoidable, poor management may increase DM losses by 60% (Borreani et al., 2018). Therefore, the use of silage bacterial inoculants is a key strategy to manipulate the fermentation process and successfully preserve forage.

As the silo reaches a state of anaerobiosis, facultative and anaerobic microorganisms proliferate and ferment the available substrate, especially sugars. Ideally, lactic acid bacteria will ferment sugars into lactic and acetic acids, reducing pH and reaching a stable silage fermentation phase (McDonald et al., 1991). However, under less-than-ideal circumstances (i.e., presence of oxygen, forages with high buffering capacity, and low substrate for bacterial fermentation), other undesirable microorganisms, such as *Clostridia*, Enterobacteria, and yeasts, may proliferate during the initial phase of silage fermentation (Pahlow et al., 2003), increasing DM losses (Borreani et al., 2018). Corn silage typically reaches a low pH rapidly, avoiding these undesirable fermentation patterns. Other widely ensiled crops such as alfalfa impose challenges for ensiling due to high buffering capacity, as well as reduced DM and water-soluble carbohydrate concentrations at harvest (Coblentz and Muck, 2012). These outcomes increase the risk of undesirable fermentation, storage losses, and decreased palatability of the silage, which impaired milk yield of dairy cows (Queiroz et al., 2018). Under these conditions, cattle producers may benefit from using homofermentative bacterial inoculants (Table 1) that have been shown to improve DM recovery (Muck and Kung, 1997; Oliveira et al., 2017) and effectively reduce silage pH while promoting rapid acidification to help curtail clostridial fermentation (Oude Elferink et al., 2001). Clostridial fermentation is associated with reduced feed consumption (Grant and Ferraretto, 2018) and adverse health events in ruminants (Driehuis et al., 2018). Homofermentative bacterial inoculants also reduce mold and *Clostridia* counts (Muck et al., 2018) and increase DM intake (**DMI**) and lactation performance by dairy cows (Oliveira et al., 2017). However, a potential downside of this type of inoculant is the reduced aerobic stability (Muck et al., 2018). Even in well-managed silages, yeasts and molds that survive anaerobic fermentation may grow rapidly and reduce aerobic stability after silo opening (Queiroz et al., 2018). Under anaerobic conditions, homofermentative lactic acid bacteria mainly produce lactic acid, which can serve as a substrate for lactate‐assimilating yeasts upon exposure to oxygen (Wohlt, 1989).

**Table 1.** Effects of microbial inoculation on fermentation profile, microbial counts, and aerobic stability of silage

	Homofermentative^1^		Heterofermentative^2^	
Item^1^	Control	Inoculated	Control	Inoculated
pH	4.10	3.99	4.13	4.19
Lactic acid, % DM	4.67	5.59	4.30	3.99
Acetic acid, % DM	1.43	1.23	1.70	2.75
Butyric acid, % DM	0.27	0.22	0.28	0.25
1,2-Propanediol, % DM	- ^3^	- ^3^	0.22	1.02
Yeast, log CFU/g	4.02	4.30	4.19	3.35
Molds, log CFU/g	3.00	2.42	2.89	2.49
Clostridia, log CFU/g	3.54	2.60	-	-
DM recovery, %	92.0	92.8	93.8	93.4
Aerobic stability, h	NS^4^	NS	111	193

^1^Adapted from Oliveira et al. (2017).

^2^Adapted from Arriola et al. (2021).

^3^Data not reported.

^4^Data reported but not significant.

Discerning changes occurring after aerobic exposure at feed-out is also critical to nutrient preservation and silage hygiene (Wilkinson and Davies, 2013; Borreani et al., 2018). Aerobic deterioration patterns are well-defined in the literature (Wilkinson and Davies, 2013). After oxygen exposure, aerobic microorganisms become active, and the aerobic deterioration process begins. Yeasts usually initiate aerobic deterioration, degrading the remaining sugars and lactic acid in the silage (Pahlow et al., 2003) and generating heat (Woolford, 1990). Utilization of lactic acid raises silage pH, allowing Bacilli and other aerobic bacteria to grow and generate more heat (McDonald et al., 1991). Mold proliferation finalizes the process of silage deterioration (Pahlow et al., 2003). According to Borreani et al. (2018), this process is exacerbated in the peripheral areas and near the sidewall of bunkers where oxygen entrapment and oxygen penetration after aerobic exposure are more pronounced. There are scenarios that further exacerbate this issue by drastically increasing yeast and mold counts. According to Buxton and O’Kiely (2003), silage harvested too dry may experience limited fermentation and greater levels of yeasts and molds. Other common scenarios, such as applying slurry on fields as fertilizer or making silage in areas prone to flooding and mud contamination while packing fresh forage, can promote exorbitant levels of yeasts and molds in forages prior to ensiling.

To estimate the negative effects of silage spoilage on farm profitability, Tabacco et al. (2011) evaluated the relationship of estimated milk yield per Mg of harvested silage DM to mold count after oxygen exposure in corn and sorghum silages. A reduction in estimated milk production was observed as mold counts exceeded 4 log CFU/g of silage. When mold counts reached or were greater than 8 log CFU/g of silage, estimated milk production was reduced by 50%. These effects were based only on the nutritive value of these forages. However, in addition to the direct effect caused by DM and nutrients losses, spoiled silage or spoiling microorganisms can also have other negative impacts on farm profitability. For example, ruminant animals may be affected by reduced palatability or have impaired performance and health (Kung et al., 1998). Some of these effects are related to the proliferation of potentially pathogenic or otherwise undesirable microorganisms in the silage (Driehuis et al., 2018), including the production of mycotoxin (Ogunade et al., 2018). Overall, the literature is consistent with multiple reports of impaired performance when spoiled silage is fed to ruminant animals. Hoffman and Ocker (1997) fed mid-lactation cows a total mixed ration (**TMR**) containing either aerobically stable or unstable high-moisture corn. Despite the lack of effects on DMI, feeding unstable silage reduced milk yield by ~3 kg/cow daily compared with cows that were fed adequate silage. Steers fed spoiled corn silage from the top area of a bunker silo had lower feed consumption and total tract organic matter and fiber digestibility (Whitlock et al., 2000).

Although the negative effects of feeding silage after prolonged aerobic exposure are undeniable, the issues extend beyond palatability and depressed intakes. Some of the mechanisms associated with impaired performance still warrant further investigation. For example, Salvo et al. (2015) fed lactating dairy cows corn silage exposed to air for 48 h or inoculated with a lactate-assimilating yeast species found in corn silage samples in Brazil (*Pichia norvegensis*, 1 × 10^5^ CFU/g of silage). Yields of 3.5% fat-corrected milk (**FCM**) were reduced for both treatments by 1.3 kg/d, on average, compared with the control treatment. However, no changes in intake were reported (Salvo et al., 2015). These effects may be partially driven by negative effects on rumen fermentation. Santos et al. (2015) inoculated ruminal fluid with three doses of a pure culture of *Issatchenkia orientalis* (previously known as *Candida krusei*) to determine the effects of low, medium, and high yeast contamination on rumen fermentation. Yeast contamination reduced NDF digestibility but did not change rumen biohydrogenation pathways (Santos et al., 2015). If a similar approach to Tabacco et al. (2011) is used with data by Santos et al. (2015), the milk/ton estimates are reduced by 40 kg/Mg in high- compared with low-yeast counts due to changes in NDF digestibility alone.

Heterofermentative bacterial inoculants are a great tool for reducing yeast and mold counts (Muck et al., 2018) and improving silage aerobic stability (Kleinschmit and Kung, 2006; Arriola et al., 2021). Main responses of inoculation of silage with heterofermentative inoculants are in Table 1. *Lentilactobacillus buchneri*, the heterofermentative strain most used in silage inoculants, improves aerobic stability by degrading moderate amounts of lactic acid and by producing 1,2-propanediol and acetic acid (Oude Elferink et al., 2001). Acetic acid is an antifungal compound that suppresses yeast and mold proliferation (Woolford, 1975). Heterofermentative bacterial inoculants can promote acetic acid production, prevent yeast proliferation, and improve aerobic stability as early as 7 to 15 d of silage storage, but these effects are more pronounced after prolonged fermentation (90 d and longer; Arriola et al., 2021).

Collectively, silage bacterial inoculants have been the most used additives to modulate silage fermentation. But commercially available bacterial inoculants differ in strains and counts, and thus, their effects on silage fermentation should be further investigated (Oliveira et al., 2017; Arriola et al., 2021), whereas utilizing research-proven commercial inoculants is advised.

## Modulating Grass Silage Fermentation with Bacterial Inoculants

Corn is the second most cultivated crop in the world, covering ~192 million ha (FAO, 2021), and it is a major source of food to humans and feed to livestock. Corn silage is a preferred feedstuff to intensively managed livestock operations and has many desirable characteristics to be preserved as silage, such as superior yield, nutritive value, and water-soluble carbohydrates concentration, and adapted to mechanized harvest and feeding. However, it is an annual crop with required background knowledge, expressive monetary investment, and is subjective to risk due to climactic variations. There are several locations that are not suitable for growing annual crops due to edaphic and climactic conditions. Grass silage can be an alternative to corn silage and it can be made from a variety of warm- and cool-season grass species, being often used in regions where growing corn is less viable or where the climate is more suited to grass crops. According to Wilkinson and Toivonen (2003), the corn silage production in the USA is 19 times greater than grass silage production (32.4 vs. 1.7 Million Mg of DM).

Despite the relatively reduced production of grass silage in the world, there has been several reports in the literature addressing management practices to improve grass silage fermentation and its nutritive value (Wing and Becker, 1963; Catchpoole and Williams, 1969; Tosi et al., 1975; Brassley, 1996). In general, grasses have undesirable characteristics to be preserved as silage, such as reduced DM concentration, reduced water-soluble carbohydrates concentration, and relatively greater buffering capacity than corn and sorghum (Sollenberger et al., 2004), but these characteristics may change significantly between cool- and warm-season grass species. Additionally, grass silage may be the only viable method of conserving forage during the growing period in regions with challenging climatic conditions.

Microbial inoculants for silage are formulations of beneficial microorganisms (typically bacteria, and sometimes fungi or yeasts) that are added to fresh forage at the time of ensiling to improve the fermentation process, preserve the nutritive value of the silage, and reduce spoilage during storage (Weinberg and Muck, 1996). These microorganisms are carefully selected to promote the growth of desirable lactic acid bacteria while suppressing the growth of unwanted microbes, such as *Clostridia*, molds, and undesirable yeasts, which can cause spoilage or poor fermentation (Muck, 2013). The most used genus of lactic acid bacteria used in silage inoculation are *Lactobacillus* spp., *Pediococcus* spp., and *Enterococcus* spp., whereas the combination of genus and strains has shown positive effects in improving silage fermentation characteristics (Kung and Ranjit, 2001; Kung et al., 2003).

Due to the widespread use of corn silage, much of the research and development of commercial microbial inoculants has focused on improving its fermentation process. These inoculants are often initially tested and optimized for corn silage and then later adapted for use in grass silage, which presents different challenges. Grass silage inoculants are not just a secondary marketing strategy but are developed with specific considerations for the characteristics of grass forage, such as its higher buffering capacity and lower water-soluble carbohydrate concentration.

Cool-season annual and perennial grasses are cultivated in subtropical and temperate regions and usually produce less forage mass compared with warm-season perennial grasses (Cooke et al., 2020). However, cool-season grasses have greater water-soluble carbohydrate concentration and less buffering capacity, which offsets the detrimental effects of reduced DM concentration compared with warm-season perennial grasses (McDonald et al., 1991). Muck et al. (2020) conducted a survey and observed that cool-season grasses had greater concentration of soluble sugars and fructan and lesser buffering capacity than warm-season perennial grasses (Table 2). Wilkinson (1990) indicated that the critical water-soluble carbohydrate concentration for successful cool-season grass silage production is 12% to 15% for grasses with DM concentration of ~20%. Positive effects of microbial inoculants in cool-season silage fermentation have been observed primarily due to the greater water-soluble carbohydrate concentration, which is a vital substrate for the lactic acid bacteria added to the silage. Wang et al. (2018) inoculated perennial ryegrass (*Lolium perenne*) and tall fescue (*Festuca arundinacea*) silage with *L. plantarum* MTD-1 and *Pediococcus acidilactic* and observed that perennial ryegrass silage treated with inoculants had lesser pH and acetic acid and greater lactic acid and in vitro DM digestibility than control (untreated) silage. Conversely, microbial inoculants did not affect the fermentation characteristics of tall fescue silage, which had greater DM concentration (29% vs. 22%) and lesser in vitro DM digestibility (53% vs. 67%) than the perennial ryegrass silage, respectively. Despite the lack of inoculant effects on tall fescue silage, the ensiled material had adequate fermentation pH and lactic acid concentrations (3.7% and 9.7%, respectively).

**Table 2.** Soluble sugars, starch, fructan, and buffering capacity of cool- and warm-season forages (Adapted from Muck et al., 2020).

Item	Cool-season grass	Warm-season grass	Temperate legumes
Soluble sugars, % DM	3–6	1–5	2–5
Starch, % DM	0–2	1–5	1–11
Fructan, % DM	3–10	-	-
Buffering capacity, mEq/kg DM	265	388	472

Warm-season perennial grasses, however, have reduced concentrations of water-soluble carbohydrates and decreased DM concentrations, usually resulting in undesirable silage fermentation. Vendramini et al. (2010) evaluated nine warm-season grass species and cultivars harvested at a 6-wk regrowth interval and observed a highly variable DM concentration, relatively high pH and decreased lactic acid concentrations in the silage of most species and cultivars tested. In addition, there was no effect of the microbial inoculant Si All (*L. plantarum, P. acidilactici,* and *E. faecium;* Grevilla Ag, Brendale QLD, Australia) on fermentation characteristics and nutritive value of the silage. Gouvea et al. (2020) evaluated seven commercial inoculants on Jiggs bermudagrass (*Cynodon dactylon*) and although there were some differences among treatments, there was minimal improvement on fermentation characteristics when treated silages were compared with the untreated control (Table 3). It is important to mention that the forage in Gouvea et al. (2020) had relatively low DM (25%) and water-soluble carbohydrate concentrations (5%), which may have led to limited conditions for appropriate activity of the microbial inoculant and desirable fermentation.

**Table 3.** Effects of microbial inoculants on fermentation characteristics (dry matter basis) of Jiggs bermudagrass (*Cynodon dactylon*) silage (Adapted from Gouvea et al., 2020).

	Inoculant^1^									
Item	Control	A	B	C	D	E	F	G	SEM	*P*-value
pH	4.86	5.06	4.95	4.86	4.90	4.96	4.79	4.80	0.09	0.10
Lactic acid, %	2.05	0.75	1.47	1.94	1.76	1.24	2.05	1.91	0.50	0.17
Acetic acid, %	1.69	2.53	2.10	1.79	2.25	1.74	1.64	1.94	0.38	0.39
Propionic acid, %	0.39	0.39	0.31	0.35	0.32	1.03	0.25	0.30	0.23	0.26
Butyric acid, %	3.37	3.76	2.99	4.17	3.55	4.31	3.66	3.59	0.82	0.62
Isobutyric acid, %	0.11	0.13	0.59	0.17	0.08	0.07	0.09	0.10	0.17	0.30
Ammonia-N, %	20.4	24.8	23.7	24.5	18.9	19.0	19.1	21.0	3.52	0.14

^1^A = *Pediococcus pentosaceous* and *Lactobacillus buchneri*; B = *Pediococcus pentosaceous* and *Propionibacteria freudenreichii*; C = *Pediococcus acidilactici, P. pentosaceus, L. plantarum,* and *L. brevis*; D = *Lactobacillus plantarum, P. acidilactici,* and *P. pentosaceus*; E = *Lactobacillus plantarum* and *P. acidilactici*; F = *Lactococcus lactis, P. pentosaceous, Enterococcus facium*, and *L. plantarum*; and G = *Lactobacillus buchneri* and *L. plantarum*.

Microbial inoculants can also yield benefits when added to silages with limited water-soluble carbohydrate and DM concentrations. Santos et al. (2014) assessed the effects of the microbial inoculant Si All C4 (*L. plantarum*, *P. acidilactici*, *and E. faecium*; Grevilla Ag) on guineagrass (*Megathyrsus maximum*) harvested a 35-, 45-, 55-, and 65-d regrowth interval. The DM and water-soluble carbohydrate concentrations ranged from 19.7% to 25.1% and 3.7% to 5.8% from 35- to 65-d regrowth interval, respectively. Microbial inoculations decreased silage pH, NH_3_-N, acetic, and butyric acid concentrations, while there was an increase in lactic acid concentration. There were no regrowth interval × microbial inoculant interactions for most response variables. Vendramini et al. (2016) observed that there was a DM concentration × microbial inoculant interaction on bermudagrass silage and wilted silage inoculated with *L. buchneri*, as silages with higher DM content (52%) had lesser lactic acid concentration than silage with lower DM content (22%).

It has been observed in silage grasses that the different abundance and diversity of epiphytic lactic acid bacteria in the pre-ensiled forage material could be an important factor determining the effectiveness of the added lactic acid bacteria from the inoculant. Jalpolsaen et al. (2022) compared the fermentation characteristics and bacterial dynamics in napiergrass (*Pennisetum purpureum*) and whole-plant corn with or without adding the microbial inoculant (*L. plantarum*, *L. brevis*, and *P. pentosaceus*). There was no effect of the inoculant in the whole-plant corn treatment and the predominant lactic acid bacteria were *Weissella*, *Enterococcus*, and *Lactococcus*, which shifted to *Lactobacillus* after ensiling. However, there was a relatively lesser abundance of epiphytic lactic acid bacteria in the napiergrass, and there was an effect of inoculation on fermentation parameters and inoculated silage had greater relative abundance of *Lactobacillus* and *Pediococcus* than the control. Besides adding desired lactic acid bacteria to the fermentation profile, the microbial inoculant may shift the fermentation profile and change the relative abundance of bacteria pool in the silage. Lazarin et al. (2024) observed that limpograss (*Hemarthria altissima*) inoculated with SiloSolve FC (*L. buchneri* LB1819 and *L. lactis* O224; Novonesis, Lyngby, Denmark) had lesser relative abundance of *Clostridium* and *Pediococcus* and greater *Lactococcus* and *Bacillus* than the untreated control. These changes in the microbial relative abundance resulted in inoculated silage with greater acetic and propionic acid and lesser butyric acid and NH_3_-N concentrations. In addition, treated silage had greater in vitro DM digestibility and CP concentrations compared with untreated silage (Lazarin et al., 2024).

In summary, while there is evidence that microbial inoculants can improve grass silage fermentation characteristics, the wide variation in chemical composition and management practices across cool- and warm-season grasses makes the outcome of inoculation less predictable. Tailored recommendations for microbial inoculant species and strains, based on forage species and management practices, may be necessary to increase the predictability of grass silage inoculation.

## Microbial Solutions for Ruminant Nutrition

Several bacterial genus, species, and strains have been listed as “Generally Recognized As Safe” (**GRAS**) for use in beef and dairy cattle rations, including *Lactobacillus* spp., *Megasphaera* spp., *Propionibacterium* spp., and *Bacillus* spp. (Krehbiel et al., 2003; McAllister et al., 2011; Luise et al., 2022). These genera have different features by which they support the health of host, and can be classified as lactic acid-producing bacteria (**LAPB**), lactic acid-utilizing bacteria (**LAUB**), as well as *Bacillus* spp. (McAllister et al., 2011; Luise et al., 2022).

### Lactic acid-producing bacteria

Among the bacterial genus that produce lactic acid, *Lactobacillus* and *Enterococcus* deserve to be highlighted. The genus *Lactobacillus* contains several species that have been fed to beef and dairy cattle, including *Ligilactobacillus animalis*, whereas *E. faecium* is present in several commercial feed additives (Shridhar et al., 2022). *Lactobacillus* spp. and *Enterococcus* spp. preferentially ferment the nutrients available in a specific media into lactate, which decreases media pH and ultimately impacts the growth and survival of potentially harmful bacteria (Kung et al., 2003; Riddel et al., 2010; Muck et al., 2018). Nonetheless, *L. animalis* has also been shown to upregulate the expression and location of tight junction proteins in the presence of challenging compounds, such as the mycotoxin deoxynivalenol (Boll et al., 2024a). Other mechanisms by which LAPB supports the health of ruminants include competitive exclusion of harmful bacteria (Riddel et al., 2010), modulation of systemic and mucosal immunity (Siepert et al., 2014), production of antimicrobial compounds (McAllister et al., 2011), and adequate microbiota balance in the presence of potentially harmful bacteria (Signorini et al., 2012).

### Lactic acid-utilizing bacteria

Excessive production and accumulation of lactic acid in the rumen can lead to digestive-related disorders such as acidosis, bloat, development of liver abscesses, and laminitis (Cooper et al., 1999; Theurer et al., 2021). Microbial solutions have been shown to alleviate ruminal accumulation of lactic acid, thus improving the health of the ruminal environment and the host (Nagaraja and Titgemeyer, 2007). Research has shown that *Megasphaera elsdenii*, *Selenomonas ruminantium*, and *Propionibacterium freudenreichii* consume lactic acid and favor propionate as an endpoint of the fermentation process (Wiryawan and Brooker, 1995; Klieve et al., 2003; Raeth-Knight et al., 2007), which increases hepatic glucose synthesis in ruminants (Bergman et al., 1990).

### Bacillus spp.

These are spore-forming gram-positive bacteria that survive and remain viable even after the feed preparation process, including pelletization, presence of macro- and trace minerals, and moisture inclusion (Bernardeau et al., 2017; Luise et al., 2022; Cappellozza et al., 2023a). Bacilli have been recognized as one of the main enzyme-producing microorganisms, given their broad range of types and amounts of enzymes produced (Schallmey et al., 2004; Luise et al., 2022). The incubation of *Bacillus licheniformis* and *B. subtilis* with forage-based substrates and TMR increased in vitro DM and NDF degradability, as well as starch degradability of starch-based ingredients (Pan et al., 2022; Cappellozza et al., 2023b). These outcomes are relevant as ruminal NDF and starch digestibility are positively associated with DMI, milk production, and milk composition in dairy cows (Oba and Allen, 1999; Ferraretto et al., 2013). Similarly, ruminal starch digestibility is positively associated with the growth performance of feedlot cattle consuming high-concentrate diets (Vander Pol et al., 2008; Owens et al., 2016).

At the lower gastrointestinal tract, *Bacillus* spp. supports the health of the host by stimulating the production of membrane-bound and secreted mucins (Santano et al., 2020), modulating the release of immunoglobulins and cytokines by immune cells located in the gut (Sun et al., 2010), competitive exclusion against potentially harmful bacteria through the formation of biofilm (Copani et al., 2020; Segura et al., 2020), promoting synthesis and release of antimicrobial and antioxidant compounds (Abriouel et al., 2011; Sumi et al., 2015; Wang et al., 2017; Luise et al., 2022), and maintaining the integrity of intestinal epithelial cells in the presence of different challenges (Rhayat et al., 2019; Boll et al., 2024b).

## Microbial Solutions for Cow-Calf Nutrition

The dietary inclusion of direct-fed microbials (**DFM**) has emerged as an alternative to antibiotics, primarily aimed at modulating ruminal fermentation, promoting the establishment of beneficial microorganisms, and enhancing both fiber and overall nutrient digestibility (Pan et al., 2022; Cappellozza et al., 2023b). Research evaluating the benefits of DFM supplementation in the cow-calf sector is surprisingly scarce, despite the forementioned benefits to overall cattle performance. Herring et al. (2000) tested the impacts of DFM supplementation to lactating *Bos taurus* Angus cows on cow milk production and composition, and body weight (**BW**) change of both cows and their calves. The DFM supplement consisted of a mixture of *Saccharomyces cerevisiae*, *E. faecium*, *L. acidophilus*, amylase from *B. subtilis*, betaglucanase from *B. subtilis* and *Aspergillus oryzae*, and hemicellulase from *B. subtilis* and *A. oryzae*. Supplementation (28.4 g/cow daily) started at 53-d postpartum (14 d before first milk production assessment) and continued for 5 periods of 14 d each. In that study, milk yield, milk crude protein concentration, and somatic cell count did not differ between cows that received or not DFM supplementation. However, DFM supplementation led to fluctuating effects on cow performance, increasing cow BW change in 2 of 5 periods but decreasing cow BW change in another 2 periods of evaluation. Ultimately, DFM supplementation did not impact cow and calf BW at weaning (Herring et al., 2000). The different composition of basal diets offered to cows throughout the study may provide an explanation for the inconsistent impacts on cow BW change and resulting lack of improvements on calf performance.

Spore-forming DFM, such as *Bacillus* spp., have been the primary microorganisms used as probiotics due to their stability and viability after processing, storage, and manipulation (Moriel et al., 2024a). Therefore, three studies using *Bos indicus*-influenced beef cattle tested the effects of spore-forming, *Bacillus*-based DFM supplementation for developing replacement heifers (Moriel et al., 2024a), pregnant heifers (Izquierdo et al., 2024a,b), and multiparous cows (Moriel et al., 2024a). In all studies, the DFM mixture consisted of *B. subtilis* and *B. licheniformis* (Bovacillus; Novonesis, Lyngby, Denmark) and was offered at a rate of 3 g per animal daily. While *Bacillus*-based DFM supplementation from weaning until the end of the breeding season did not impact growth performance, it increased the percentage of pubertal heifers and enhanced their reproductive tract score at the start of the breeding season compared with no DFM supplementation (Moriel et al., 2024a). Moreover, year-round access to a *Bacillus*-based DFM mixture incorporated into trace mineral supplementation for multiparous beef cows (and their calves) led to increased overall trace mineral supplement intake, greater gains in cow body condition score (**BCS**) during the breeding season, and heavier calf weaning weights compared with cows without DFM supplementation (Moriel et al., 2024a). These results highlight the potential of DFM supplementation to enhance the overall cow-calf productivity.

Nutritional management of beef cows during gestation and early lactation has been linked to improved offspring growth performance and immune response following birth (Moriel et al., 2021). The mammalian gastrointestinal tract is sterile in utero, but it undergoes rapid microbial colonization after birth (Fanaro et al., 2003), which is essential for the development of the gut and immune system (Hansen et al., 2012). This colonization of the neonatal gut can be influenced by both maternal and offspring diet (Fouhy et al., 2012; Rodriguez et al., 2015). For instance, Izquierdo et al., (2024a,b) assessed the effects of maternal supplementation with a *Bacillus*-based DFM during both pre- and postpartum periods on physiology, immune function, and growth performance of their *B. indicus*-influenced beef offspring, along with evaluating both maternal and offspring metabolome. Briefly, fall-calving, Brangus crossbred beef heifers (20 to 22 mo of age) pregnant with their first offspring were allocated into bahiagrass pastures and randomly assigned to receive 1 kg/d of soybean hulls added (**BAC**) or not (**CON**) with DFM containing *B. subtilis* and *B. licheniformis*. Treatments were provided from days 0 to 242 (139 ± 4 d prepartum until 104 ± 4 d postpartum). Calves did not have access to maternal supplements. All calves were weaned on day 242 (96 ± 30 d of age), acclimated to the stress of weaning for 16 d, and then allocated into drylot pens and fed the same concentrate DM at 3.25% of BW for 60 d (days 258 to 318).

Precalving BCS change and resulting BCS at calving were greater for BAC vs. CON heifers (Table 4; Izquierdo et al. 2024a), suggesting that the inclusion of *Bacillus*-based DFM increased forage DM and NDF digestibility (Pan et al., 2022). Despite greater BCS at calving in BAC heifers, final pregnancy rates did not differ between CON and BAC heifers (88.6% vs. 88.9%, respectively; Izquierdo et al., 2024a), likely due to optimal BCS at calving in both groups (Moriel et al., 2024b) and the use of early weaning to enhance pregnancy rates (Arthington and Kalmbacher, 2003). Nonetheless, maternal nutrition during gestation often unleashes a cascade of modifications to animal metabolism (Moriel et al., 2021). Supplementing BAC increased maternal prepartum plasma concentrations of glucose (Izquierdo et al., 2024a), which is essential for fetal growth (Bauer et al., 1998). Supplementing BAC also increased maternal plasma concentrations of 17 metabolites compared with CON heifers, including phosphatidylcholines and amino acids-related classes (Izquierdo et al., 2024b). Collectively, these data indicate that *Bacillus*-based DFM supplementation during pregnancy improved the energy and protein metabolism in heifers (Izquierdo et al., 2024b), leading to greater prepartum BCS gain compared with no DFM supplementation (Izquierdo et al., 2024a).

**Table 4.** Maternal and offspring performance following maternal supplementation with soybean hulls added (**BAC**) or not (**CON**) with a mixture of *Bacillus*-based DFM (3 g/cow daily) from days 0 to 242 (adapted from Izquierdo et al., 2024a).

	Maternal treatment^1^			
Item	CON	BAC	SEM	*P*-value
Heifer body condition score				
Day 0	6.00	6.05	0.083	0.63
Day 91 (near calving)	6.09	6.37	0.083	0.01
Day 242 (start of the breeding season)	5.04	5.04	0.083	0.95
Day 312 (end of the breeding season)	5.20	5.25	0.083	0.66
Heifer body condition score change				
Day 0 to 91	0.05	0.36	0.102	0.05
Day 91 to 242	−1.06	−1.33	0.167	0.28
Day 242 to 312	0.17	0.23	0.099	0.69
Calf body weight, kg				
Birth	28.1	29.5	0.99	0.34
Day 242 (weaning)	81	81	3.1	0.98
Day 258 (drylot entry)	88	89	3.1	0.84
Day 319 (drylot exit)	149	158	3.1	0.05
Calf drylot performance (Day 258 to 319)				
Average daily gain, kg/day	1.01	1.11	0.035	0.04
Total dry matter intake, % of body weight	3.24	3.27	0.038	0.73
Gain:feed	0.25	0.27	0.004	0.05
Calf serum BRSV titers, log_2_				
Day 271	1.2	1.43	0.363	0.65
Day 287	1.63	2.5	0.363	0.04
Day 319	3	2.53	0.363	0.36
Seroconversion to PI-3, % of total				
Day 271	80	93.3	3.57	<0.01
Day 287	100	100	3.57	0.99
Day 319	100	100	3.57	0.99

^1^Maternal treatments were provided on average for 139 ± 4 d prepartum and 104 ± 4 d postpartum (days 0 to 242). Calves did not have access to maternal supplements and were weaned on day 242 at 96 ± 30 d of age. On day 258, calves were transferred to drylot pens and were limit-fed the same concentrate at 3.25% of BW until day 319. On day 271, each calf was dewormed (Safe-guard, Merck Animal Health, Summit, NJ) and vaccinated against pathogens associated with BRD (Bovi Shield Gold One Shot; Zoetis Inc., New York, NY) including *bovine respiratory syncytial virus* (**BRSV**) and *parainfluenza-3 virus* (**PI-3**) and *Clostridium* (Ultrabac 7, Zoetis Inc.). On day 287, each calf received booster vaccinations of Bovi Shield Gold 5 and Ultrabac 8 (Zoetis Inc.).

Izquierdo et al. (2024a) also reported that maternal BAC supplementation had no effect on calf birth and weaning weights but improved average daily gain and feed efficiency during the drylot phase, resulting in greater BW at drylot exit compared with calves from CON cows (Table 4; Izquierdo et al., 2024a). Maternal BAC supplementation also improved the vaccination-induced humoral immune function in the offspring (Table 4), increasing production of serum titers against bovine respiratory syncytial virus and increasing positive seroconversion against parainfluenza virus (Izquierdo et al., 2024a). This improved immune function in the offspring has consistently been reported following improved maternal nutrition during the third trimester of gestation (Moriel et al., 2021). Calves did not have access to maternal supplements. Hence, both improved growth and immune function of calves were caused by the greater maternal prepartum BCS gain (Marques et al., 2016; Moriel et al., 2024b), the altered maternal physiology (Moriel et al., 2021), and the resulting calf metabolome (Izquierdo et al., 2024b) from maternal supplementation of BAC. For instance, calves born from heifers offered BAC exhibited enrichments of multiple pathways associated with amino acid metabolism (particularly methionine) and regulation of innate and humoral immune function compared with cohorts born from CON heifers (Izquierdo et al., 2024b).

Recent advancements in DFM supplementation, particularly *Bacillus* spp., have shown promise for enhancing cow-calf productivity through improved gut health, immune function, and nutrient metabolism. Initial findings from limited studies on *B. indicus*-influenced cattle suggest that *Bacillus*-based DFM may enhance reproductive outcomes and BCS of beef females, as well as improve calf growth and immune response following DFM supplementation in utero. These results underscore the potential of DFM to benefit cow-calf operations and warrant further exploration. Despite this research progress, there remains a scarcity of research on DFM effects in cow-calf systems, especially across different DFM mixtures and dosages, cattle subspecies and breeds, environments (i.e., temperate vs. tropical/subtropical), and nutritional strategies (i.e., warm-vs. cool-season forages).

## Microbial Solutions for Forage-Fed Growing Beef Cattle

Backgrounding programs are essential management systems in beef production designed to prepare weaned calves for feedlot finishing (Wieringa et al., 1974; Cox-O’Neill et al., 2017; Hall et al., 2018). These programs aim to achieve moderate, steady growth by emphasizing forage-based, cost-effective diets, and incorporating management strategies that cattle improve health, adaptability, and performance (Hilton, 2015). Hence, the backgrounding phase provides an opportunity to implement nutritional practices that prepare cattle to withstand the stressors associated with feedlot entry, promoting animal growth, welfare, and productivity across beef production sectors (Roeber et al., 2001; Duff and Galyean, 2006). During the initial weeks of backgrounding, however, the elevated stress associated with weaning and management factors such as marketing, commingling, and road-transport leads to inadequate nutrient intake and immunosuppression (Hutcheson and Cole, 1986; Duff and Galyean, 2006; Richeson et al., 2019). One nutritional strategy to stimulate feed intake and immunity is the use of *Bacillus*-based DFM, which has been widely studied for their potential to enhance beef cattle health and performance while addressing public concerns over antibiotic use (Krehbiel et al., 2003; Hall et al., 2018).

**Figure 1.**
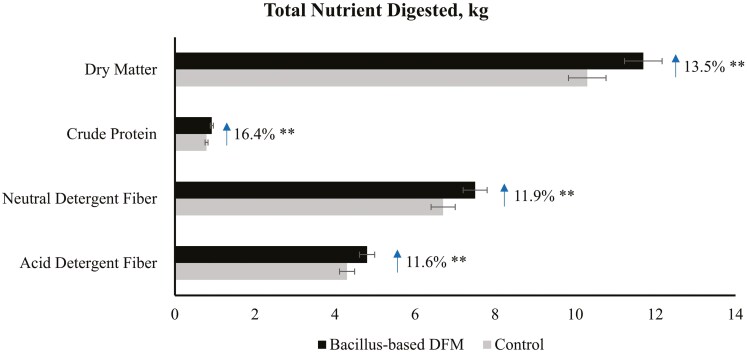
Total nutrient digested of rumen-cannulated beef cows fed with forage-based diets and supplemented or not (**CON**) with *Bacillus*-based direct-fed microbials (**DFM**). Adapted from Marques et al. (2024); **P < 0.01.

Recent studies have demonstrated that *Bacillus*-based DFM can enhance feed efficiency (Dias et al., 2022; Cordeiro et al., 2024), improve in vitro digestibility of DM, NDF, and starch (Pan et al., 2022; Cappellozza et al., 2023b), and bolster health and immunity (Mackey et al., 2024) in growing cattle. Additionally, *Bacillus* spp. can colonize the rumen and intestine, affecting digestion at all sites (Green et al., 1999; Fuerniss et al., 2022) due to their ability to synthesize digestive enzymes (Schallmey et al., 2004; Elshaghabee et al., 2017; Luise et al., 2022). Marques et al. (2024) evaluated the effects of *Bacillus-*based DFM supplementation (Bovacillus; Novonesis) on DMI, ruminal degradability, total apparent nutrient digestibility, and ruminal fermentation parameters of rumen-cannulated cows fed a forage-based diet. Supplementing the *Bacillus*-based DFM to cows consuming a forage-based diet enhanced the total amount of DM, CP, NDF, and ADF digested by 13.5%, 16.4%, 11.9%, and 11.6%, respectively (Figure 1). These findings align with Pan et al. (2022), who demonstrated that combining *B. licheniformis* and *B. subtilis* improved in vitro DM and NDF digestibility in 8 out of 10 forage sources (Table 5). Cappellozza et al. (2023b) also reported that in vitro NDF digestibility was improved by 12.8% after 24-h incubation with a *Bacillus*-based DFM, whereas in vitro DM digestibility was also increased by 6.5% after 48-h incubation (Cappellozza et al., 2023b). These results highlight the potential of using *Bacillus*-based DFM to enhance forage digestibility and intake in backgrounding programs.

**Table 5.** Mean in vitro and in vivo dry matter, and neutral detergent fiber digestibility of forage-based sources added or not (**CON**) a *Bacillus*-based direct-fed microbial (**DFM**).^1^

Item	CON	DFM	Improvement, %
Digestibility in vitro			
Pan et al. (2022) ^2^			
Dry Matter, %	44.6	51.2	11.5
Neutral Detergent Fiber, %	30.4	38.6	27.0
Cappellozza et al. (2023b)			
Dry Matter, %	57.0	59.1	3.7
Neutral Detergent Fiber, %	39.2	45.1	15.2
Digestibility in vivo			
Marques et al. (2024)			
Dry Matter, %	70.4	72.8	3.4
Neutral Detergent Fiber, %	6.7	7.5	11.9

^1^Results were adapted from published articles.

^2^Average digestibility of 8 forages using in Pan et al. (2022): ryegrass (*Lolium* spp.), alfalfa hay (*Medicago sativa*), leucaena (*Leucaena leucocephala*), corn silage (*Zea mays*), spinifex (*Geophaps plumifera*), buffel grass (*Cenchrus ciliaris*), Mitchell grass (*Astrebla*), and rhodes grass hay (*Chloris gayana*). Digestibility was analyzed at 24 and 48 h post-DFM inoculation.

As previously mentioned, reduced feed intake during the initial weeks of pasture- or drylot-based backgrounding programs is a major challenge that needs to be addressed (Hutcheson and Cole, 1986; Senn et al., 1996; Burgos et al., 2001). Cattle typically consume 0.5% to 1.5% of their BW during the first week upon arrival, increasing to 1.5% to 2.5% of BW in the second week. Intake levels of 2.5% to 3.5% BW are often reached by the third to fourth week upon arrival (Hutcheson and Cole, 1986). These changes in intake are accompanied by disruptions in the ruminal microbiota, which may reduce fermentation capacity and subsequently decrease feed intake (Galyean et al., 1981; Cole and Hutcheson, 1985; Cole et al., 1986). Therefore, enhancing nutrient intake, digestibility, and degradability in forage-fed backgrounding cattle is a key priority for improving their performance and health. *Bacillus*-based DFM may be particularly effective in diets with high fiber content, likely due to the proteolytic and fibrolytic enzyme production (Schallmey et al., 2004). Additionally, *B. subtilis* produces expansin-like proteins, notably BsEXLX1 (Pech-Cervantes et al., 2019), which play a critical role in cellulose degradation by interacting through their binding domain, inducing glucose chain twisting, and disrupting hydrogen bonds (Silveira and Skaf, 2016). Pan et al. (2022) suggested that these enzymes and expansin-like proteins may synergistically enhance fiber digestibility (Table 5). Accordingly, Marques et al. (2024) reported improved nutrient degradability and digestibility of cows supplemented with a *Bacillus*-based DFM, resulting in a 4.5% increase in DMI. Dietary DFM have been shown to enhance the cellulolytic bacterial population in the rumen (Harrison et al., 1988; Frumholtz et al., 1989; Dawson et al., 1990). While both in vitro and in vivo studies suggest positive effects on rumen activity and microbial populations, these findings do not consistently result in improved ruminal fermentation or volatile fatty acid (**VFA**) production (Martin and Nisbet, 1992). Marques et al. (2024) observed no improvements in ruminal fermentation parameters in rumen-cannulated cows fed a forage-based diet supplemented with *Bacillus*-based DFM. Nevertheless, the potential of DFM to positively influence rumen microbiota and overall animal health presents a promising avenue for optimizing ruminant nutrition and warrants further exploration to fully realize their benefits in backgrounding programs.

Newly received background cattle are often classified as high-risk because of the multiple stressors that they are exposed in a short period of time (Cooke et al., 2017; Wilson et al., 2017). These concurrent stressors significantly impact physiological and immune responses, causing immunosuppression and increasing risk of bovine respiratory disease (**BRD**; Sweiger and Nichols, 2010; Cooke, 2017). Recent nutritional interventions, such as *Bacillus*-based DFM, have been developed to enhance the performance and immunity in high-risk cattle (Galyean et al., 2022). Supplementing a *Bacillus-*based DFM to high-risk receiving steers in a drylot-based background program reduced mortality and the number of antimicrobial treatments to BRD (Colombo et al. 2021) and alleviated the incidence of liver abscesses when fed until slaughter (Mackey et al., 2023). Rhayat et al. (2019) also reported that *Bacillus*-based DFM alleviated inflammatory responses in the Caco-2 intestinal cells while strengthening the gut barrier.

**Figure 2.**
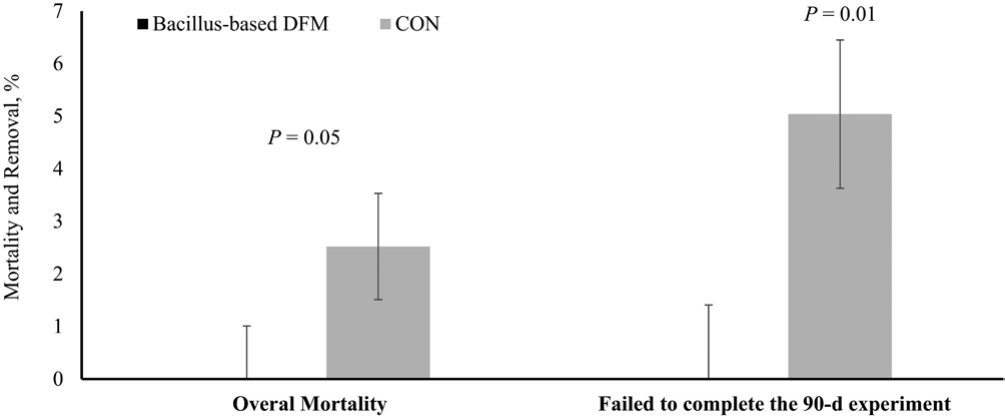
Mortality and removal percentage of high-risk stocker steers supplemented or not (**CON**) with *Bacillus*-based direct-fed microbials (**DFM**) during a 90-d grazing period. Adapted from Mackey et al. (2024); steers failed to complete the 90-d experiment include mortalities and extreme morbidity.

**Figure 3.**
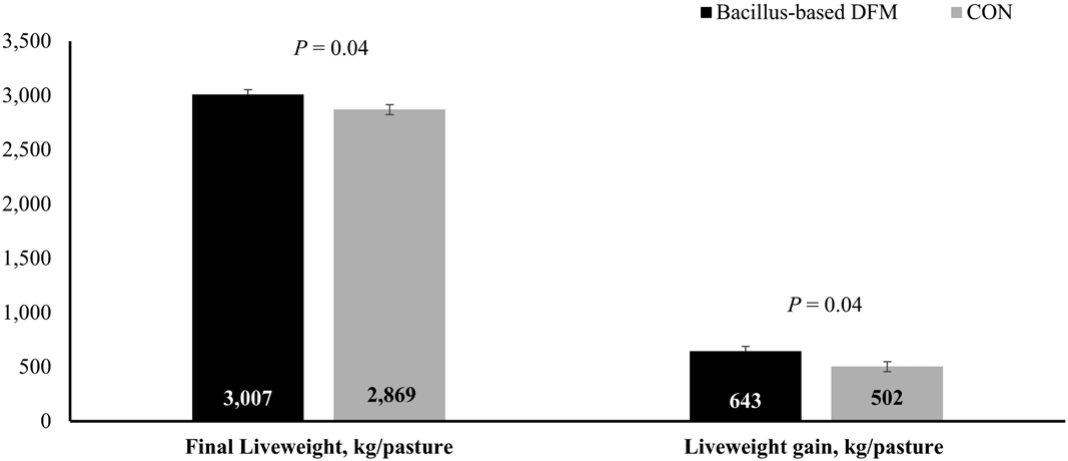
Performance parameters of high-risk stocker steers supplemented or not (**CON**) with *Bacillus*-based direct-fed microbials (**DFM**) during a 90-d grazing period. Adapted from Mackey et al. (2024).

To capture the stress and health challenges faced by high-risk cattle in a forage-based backgrounding program, Mackey et al. (2024) evaluated steers purchased from a commercial auction yard, transported to a research facility, and processed into a grazing system within 48 h. Steers were assigned to receive or not a *Bacillus*-based DFM (Bovacillus; Novonesis) during a 90-d grazing period. Overall, supplementing steers with the *Bacillus*-based DFM did not improve growth rates or reduce the incidence of BRD. However, none of the steers that received the *Bacillus*-based DFM died from BRD or were removed from the study for health reasons, whereas 5% of the nonsupplemented steers failed to complete the 90-d study (Figure 2). The causes for study removal of nonsupplemented steers were severe toe tip necrosis syndrome, severe infectious bovine kerato-conjunctivitis, and unspecified neurological disorder, in addition to mortality due to BRD, although it is unclear if steer removal due to these disorders were mitigated by BOV supplementation (Mackey et al., 2024). Steers supplemented with the *Bacillus-*based DFM also exhibited less plasma haptoglobin concentrations during the first 28 d of the 90-d grazing period, indicating reduced stress- and/or pathogen-induced inflammation. Consequently, pasture-based liveweight production increased by 28% with *Bacillus*-based DFM supplementation (Figure 3). These findings suggest that supplementing a *Bacillus*-based DFM offers a promising alternative for improving the health and overall productivity of high-risk stocker cattle. Incorporating a *Bacillus*-based DFM into backgrounding and feedlot receiving programs appears to be an alternative to improve forage intake, digestibility, total degradability of nutrients, health, and productivity of beef cattle (Colombo et al., 2021; Mackey et al., 2024).

## Microbial Solutions for Feedlot Cattle

While the original purpose of DFM use in beef cattle was to enhance rumen health and to support a healthy gastrointestinal tract microflora in young calves, DFM use in finishing cattle has been steadily increasing (LeJeune and Wetzel, 2007). The reason for this increase in use of DFM cattle diets is primarily due to public health concern with antimicrobial resistance from the use of antimicrobial technologies in feedlot cattle diets, and to improve performance and reduce the incidence of adverse health events. Feedlot cattle consuming high-concentrate diets for extended periods of time can experience shifts in gastrointestinal tract microflora that may be detrimental to ruminal or intestinal health, which can impact overall animal health and performance (Theurer et al., 2021). There are several species and strains of DFM (e.g., LAPB and LAUB) that can optimize the microbial populations within the gastrointestinal tract to improve gut health (Miller, 2024). With improved gut health, improvements in nutrient digestion, absorption, immune function, and intestinal epithelial barrier function are possible. Several recent experiments supplementing feedlot cattle with DFM have reported improved ADF digestibility, increased ruminal pH, improved ruminal lactic acid clearance, inhibited in vitro growth of *C. perfringens* types A and C, increased superoxide dismutase activity in red blood cells, and improved average daily gains during certain portions of the feeding period (Guimaraes et al., 2024; Lopez et al., 2024; Miller et al., 2024; Silva et al., 2024). However, other DFM supplementation experiments have not reported improvements in growth and health responses in feedlot cattle (Keyser et al., 2007; Mayer et al., 2022; Gubbels et al., 2023; Thompson et al., 2025). The reason for discrepancies between experiments may be due to differences in animal production scenarios (i.e., receiving vs. finishing cattle), the ingredient and nutrient composition of the basal diet (i.e., type of dietary forage and grains), dosage of the DFM, and the bacterial composition of the DFM.

Numerous studies have been conducted to evaluate the effectiveness of a large variety of bacterial-based DFM products and have been summarized by Miller (2024). Briefly, while the amount of variation in bacterial DFM products used is large, many studies have reported positive results from the utilization of these products. Earlier research by Gill et al. (1987) reported that feeding a bacterial DFM in the 28-d receiving period of feedlot cattle resulted in a 9.5% improvement in feed efficiency, a 10.9% reduction in morbidity, and a 9.3% improvement in average daily gain. Overall, these results demonstrate improved animal performance during one of the most stressful times for feedlot cattle. Other researchers used a DFM containing *L. acidophilus* and *P. freudenreichii* to evaluate morphology of the digestive tract of neonatal Holstein calves (Dick et al., 2013). These authors reported greater average ileal villus height, crypt depth, and total (villus + crypt) height before weaning and greater average ruminal papillae width in DFM-supplemented animals. No differences in final ADG or BW were reported when comparing DFM-fed and control calves. While normal performance measures of the calves were not improved in Dick et al. (2013), the overall development of the digestive tract was improved in DFM-supplemented calves.

A DFM product using a combination of *E. faecium* (6 × 10^9^ CFU/d) and yeast resulted in increased propionate and decreased butyrate concentrations when fed to feedlot cattle receiving a high-concentrate diet (Beauchemin et al., 2003). This same study also reported a tendency for increased flow of nitrogen from feed into the duodenum, while microbial nitrogen tended to decrease. These researchers stated that besides the propionate increase, these other metabolic changes could be seen as undesirable (Beauchemin et al., 2003). Other researchers evaluated different bacterial strains on 30 rumen-fistulated steers from the Nellore breed being fed a high-concentrate diet, using the combination of *E. faecium* and *Saccharomyces cerevisiae* at 3.5 × 10^9^ CFU/g or a combination of *B. licheniformis* and *B. subtilis* at a concentration of 3.2 × 10^9^ CFU/g (Dias et al., 2022). These researchers reported that DFM supplementation, regardless of combination, reduced rumen NH_3_-N concentrations and acetate:propionate ratio and tended to improve the performance of steers when compared with nonsupplemented cattle. In contrast to these studies, others have reported no performance responses to DFM supplementation for feedlot receiving cattle (Kiesling and Lofgreen, 1981; Krehbiel et al., 2001) or newly weaned calves (Drew and Thomas, 1981; Kercher et al., 1985, 1986). Additionally, no treatment effects on performance parameters were reported when *M. elsdenii* was fed at the beginning of the feedlot period to *B. indicus* bulls (Lopes et al., 2021).

Food safety has also been one point of interest when bacterial-based DFM are fed to finishing beef cattle. More specifically, *Salmonella enterica* has been identified as one of the most important and prevalent food-borne pathogens, resulting in ~150,000 deaths per year (Majowicz et al., 2010; Das et al., 2013). Although food inspection protocols have been improving over time, the fact that *Salmonella* resides in the lymph nodes and these cannot be removed from the carcass complicate this matter. Therefore, alternatives that reduce the presence of *S. enterica* into the lymph nodes and, therefore, the carcass will be beneficial to the entire production chain and for the human population consuming such edible products. Previous authors demonstrated that incubating mycotoxin with *L. animalis* resulted in an upregulation of tight junction protein when compared with the mycotoxin alone (Boll et al., 2024a). In beef calves, the feeding of *L. animalis* and *P. freudenreichii* reduced the proportion of animals positive for *S.* Typhimurium and improved the health scores of the animals post-*Salmonella* challenge (Cull et al., 2022). Lastly, feeding the same combination as aforementioned at the dose of 1 × 10^9^ CFU/head per day reduced the prevalence and counts of *Salmonella* in lymph nodes (Vipham et al., 2015), suggesting that this bacterial-based DFM was possibly: 1) supporting the integrity of the intestinal epithelial cells, 2) inhibiting the activity of this potentially harmful bacteria, or 3) a combination of both. Based on these data, Gresse et al. (2024) designed a series of experiments to evaluate the effects of adding a multispecies bacteria-based DFM on in vitro *S. enterica* invasion (Exp. 1), inhibition (Exp. 2), and in the support of intestinal epithelial cell monolayers challenged with *Salmonella* (Exp. 3).

In Exp. 1, HT29-MTX-E12 cells were grown for 14 d and incubated or not with a bacterial-based DFM containing *L. animalis*, *P. freudenreichii*, *B. licheniformis*, and *B. subtilis* at 1 × 10^8^ CFU/well for 30 min prior to the addition of 1.41 × 10^8^ CFU of *S.* Typhimurium per well. Ninety minutes later, cell lysis was performed and *S.* Typhimurium was counted to determine the proportion of cells invading the epithelial cells and that remained viable. Adding the bacterial-based DFM reduced the invading *Salmonella* cells by 90.8% while reducing its viability by 21.0%, suggesting that the DFM works to support the integrity of the cells, but also by inhibiting the growth of this potentially harmful bacteria. In fact, the latter result was supported by the data generated in the agar-diffusion assay (Exp. 2), as the zone of inhibition of the DFM against *S.* Dublin averaged 8.8 mm. In the last set of experiments (*n* = 3), the transepithelial electrical resistance (**TEER**) assays were performed. Firstly, all DFM doses improved the integrity of the cells, but a dose-response was observed such that adding 6.25 × 10^6^ CFU/well of the DFM yielded a better TEER overtime when compared with the 10^7^ or 10^8^ doses. These results might be attributed to the fact that under homeostasis, the pH reduction caused by the greater DFM doses likely impacted the integrity of the cells when compared with the lower dose (10^6^ CFU/well). However, when *S.* Typhimurium was added into the assay, all doses alleviated the negative effect of the challenge, whereas in the presence of hydrogen peroxide as a stressor, the dose of 1 × 10^8^ CFU/well supported the integrity of the cells for a longer period of time when compared with the other doses. Altogether, these results demonstrate that the multispecies bacterial-based DFM were an effective alternative to counteract the potentially damaging effects of *Salmonella*, supporting the integrity of the intestinal epithelial cells under in vitro settings. Nonetheless, in vivo studies are warranted to evaluate such effects and possible benefits in cattle fed the DFM until slaughter.

## Microbial Solutions for Dairy Cattle

Enhancing feed efficiency in dairy cattle is important because it not only improves the utilization of natural resources and reduces the environmental impact associated with milk production but also contributes to improve economic returns in dairy systems (VandeHaar et al., 2016; Bach et al., 2020). Key factors modulating feed efficiency of cows include 1) the fermentation process in the rumen, 2) the digestion in both the small and large intestines, 3) the way nutrients are metabolized, 4) the partition of these nutrients into different body functions, and 5) the genetics of the host (which, to some extent, affects most of the previous factors). These five aspects are influenced by the type and amounts of dietary nutrients, which in turn influence the rumen, as well as intestinal microbiomes, and thus, feed efficiency can partially be modulated by DFM. Nevertheless, maximizing feed efficiency should not be the sole focus of dairy production as one of the most relevant objectives should be maximizing income over feed cost, or the gross margin of milk production after accounting for feed costs. On some occasions, the investment made to improve feed efficiency or milk production is not compensated for by the returns from milk sales. This is partly because cows are fed in groups and not all cows within a group may respond with improved feed efficiency or increase milk yield (due to differences in lactation stage, genetics, and other factors) when fed a diet with a greater nutrient density, or when supplementing with a specific additive (Bach, 2023). Thus, when assessing the performance response to DFM, it is important to properly define the target animal (i.e., parity, stage of lactation, level of production, feed consumption level) and the type of ration offered (i.e., nutrient profile and composition). There is evidence in the literature that the genome of the host exerts an influence in the rumen microbiome (Abbas et al., 2020; Zhang et al., 2020) and that this relation is partly responsible for the feed efficiency of the cow (Martinez-Boggio et al., 2024). Thus, when attempting to modify the rumen (or the intestinal) microbiome, nutritionists may encounter some resistance to change due to the genetic background of cows and type of dietary profile (i.e., energy and starch density). Nevertheless, several studies have shown that the ruminal (Delgado et al. 2019; Martinez-Boggio et al., 2024) and the hindgut microbiome (Bach et al., 2019; Monteiro et al., 2022) are correlated with feed efficiency in dairy cattle.

One of the most important factors influencing the gut microbiome is the type and amount of nutrients consumed by the cow. Most DFM for dairy cows have focused on the rumen to foster fiber degradation or reduce risk of rumen acidosis and have mainly been yeast-based. But there is a poorly explored opportunity to use bacterial-based DFM targeting not only the rumen but also the small and large intestines in lactating cows. The number of bacteria in the rumen is about 10^10^ to 10^14^ cells/mL (O’Hara et al., 2020), which interestingly is equivalent to the abundance of bacteria found in the large intestine of cattle, especially in the colon (Imai et al., 1981). Furthermore, the small intestine, which in ruminants tends to be obviated, contains about 10^4^ bacterial cells/mL (Frey et al., 2010). The bacterial populations in the rumen, small, and large intestine are quite distinct and can be grouped in well-differentiated clusters (Mao et al., 2015). As expected, the microbial profile of the rumen changes much more than that of the intestine across lactation (Monteiro et al., 2022), probably because the rumen microbiome is more dependent on the ration consumed by the animal, whereas the nutrient profile that reaches the intestine is more constant.

As previously mentioned, several bacteria-based DFM have been listed as GRAS, but present different features by which they may support health and promote improvements in feed efficiency. In adult dairy cattle, most of these DFM are targeting the rumen, while just a few have been tested for their potential activity in the intestine of adult cattle, although perhaps using DFM to target the intestine could have a more consistent animal response because dietary perturbations attenuate as the distance from the rumen diminishes (Bach et al., 2019). Nevertheless, inconclusive results are reported in the literature when supplementing DFM, mainly due to the type of DFM supplemented and the lack of a holistic approach to understand the potential alteration in the dynamics of the entire digestive microbiome.

### Yeast-based DFM

Live yeast DFM supplementation in dairy cattle is commonly justified to achieve a change in rumen fermentation toward improved fiber digestion and increased rumen pH. Most yeast are unable to proliferate in the absence of oxygen, although there are some yeasts, such as strains of *Pichia* that dived in anaerobiosis and are found in the rumen of cows. Another yeast able to multiply in anaerobiosis is *S. cerevisiae*, although this species is not part of the natural rumen microbiome, it is capable of growing in the rumen provided that there is ergosterol produced by other bacteria (Krause, 2023). Due to its relative easiness of cultivation, it has been extensively used in ruminants. Newbold et al. (1996) reported an increased in vitro oxygen removal from rumen fluid when adding 1.3 mg/L of *S. cerevisiae*. Chaucheyras et al. (1996) reported that adding 10^7^ CFU of yeast /mL of culture medium along with an equivalent protein mass of live *Streptococcus bovis* in the presence of glucose reduced the production of lactic acid because yeast was able to outcompete *S. bovis* for the use of glucose. Several studies have also reported that live yeast supplementation in vitro stimulates L-lactate utilization by fostering the growth of *M. elsdenii* and *S. ruminantium* (Nisbet and Martin, 1991; Pinochle et al., 2013). However, the doses used in vitro seem extremely high compared with doses used in vivo. For example, to achieve the 1.3 mg/L of *S. cerevisiae* used by Newbold et al. (1996) in vitro, lactating cows would be supplemented about 300 g of yeast, while in practice, they typically are supplemented <5 g. Similarly, the stimulation of *M. elsdenii* and *S. ruminantium* was achieved when reaching concentration when using 2.5 g of yeast/L (authors did not report CFU), which are doses that could not be achieved in vivo unless large amounts (~500 g) of yeast were fed.

Nevertheless, there are some reports in vivo showing positive responses to yeast supplementation in dairy cattle at much lower doses than those used in vitro. Under conditions of heat stress, Moallem et al. (2009) found an increase in DMI and a consequent improvement in milk yield when supplementing dairy cows with 2 × 10^11^ CFU/d of live yeast, but no changes were observed in digestibility or rumen pH. Also, under heat stress, Li et al. (2022) reported an increase in DMI and milk yield in dairy cows supplemented with 2 × 10^11^ or 4 × 10^11^ CFU of live yeast/d. Under thermoneutral conditions, Meller et al. (2014) reported a modest, but significant, increase in milk yield in early lactation cows. Dias et al. (2018) supplemented dairy cows with 15 g of live yeast/d (CFU was not reported) and described increases in milk yield and rumen pH with no changes in DMI. Similarly, Williams et al. (1991) found an increase in energy-corrected milk when supplementing dairy cows with 5 × 10^10^ CFU/d of *S. cerevisia*e, especially when feeding a 40:60 vs. a 50:50 forage:concentrate ration. Other studies have reported increases in total tract digestibility of DM with no consequences on milk yield (Ferraretto et al., 2012) when supplementing 3 or 6 × 10^10^ CFU/d of live yeast, but this increase in digestibility could have been partly due to a lower NDF intake in supplemented cows. Moreover, supplementation of dairy cows with 5 × 10^11^ CFU of live yeast/d (Ferreira, 2019) did not improve milking performance or total tract digestibility of DM and nutrients.

The potential effect of live yeast on improving rumen pH described in vitro has been observed in few in vivo studies (Williams et al., 1991; Bach et al., 2007) using doses of 5 and 1 × 10^10^ CFU/d, respectively. Chung et al. (2011) evaluated the effects of two live yeast strains on rumen fermentation in vivo, and reported either no change with a strain previously reported to elicit an increase in rumen pH (Bach et al., 2007; Thrune et al., 2009) or a reduction in rumen pH with a strain previously reported to increase fiber degradation in vitro (Chaucheyras-Durand et al., 2008). But other studies have reported no changes in rumen pH when supplementing ~10^11^ CFU of live yeast/d (Ambriz-Vilchis et al., 2017; Cattaneo et al., 2023). Pinochle et al. (2013) reported an increase in rumen pH due to the reduction of lactic concentrations in the ruminal fluid along with lower redox potential and a shift in enumeration of *Ruminococcus* and *Fibrobacter* genera when supplementing cows with 5 × 10^9^ CFU/d. However, Pinochle et al. (2013) did not evaluate DMI; thus, changes in rumen fermentation could be confounded with potential treatment effects on feed intake. More recently, there has been an interest in evaluating the role of live yeast during the transition period with cows being supplemented both before and after calving, although results are still inconclusive. Bach et al. (2019) supplemented 1 × 10^10^ CFU of live yeast for 21 d pre- and postpartum and reported an increase in milk yield which was more closely associated with the colon microbiome and the DMI before calving than after calving, as DMI during lactation was not influenced by live yeast supplementation. Similarly, Cattaneo et al. (2023) reported an increase in DMI and a tendency toward improved milk yield at week 5 of lactation along with no changes in rumen pH in Holstein cows that received 10^11^ CFU of live yeast/d for 21 d before and 21 d after calving. Under heat stress conditions, Nasiri et al. (2019) reported an increase in DMI pre-, but not postpartum, and described an increase in milk yield when supplementing 6 × 10^10^ CFU of yeast/d to dairy cows during the transition period.

Some studies have also looked at the potential impact of *S. cerevisiae* on the hindgut microbiome. Bach et al. (2019) reported that *S. cerevisiae* supplementation before calving affected the microbiome in the hindgut and these changes were associated with future milking performance. More recently, Li et al. (2022) reported a reduction in relative abundance of *Bacteroides* and *Oscillospirales* in the feces of dairy cattle (which was used as proxy of the hindgut microbiome) supplemented with roughly10^11^ CFU of live yeast/d. However, the potential mechanisms by which yeast-based DFM may alter the microbiome of the hindgut are not fully known. One hypothesis is that some of the cell components of live yeast may reach the intestine and serve as nutrients to stimulate the growth and activity of some bacteria, as feeding inactivated live yeast has also been shown to, in some instances, alter the hindgut microbiome in cattle (Jiang et al., 2023).

Part of the variable response to live yeast describe herein is due to the strain (Chaucheyras et al., 2008), the type of ration (Dias et al., 2018; Ferreira et al., 2019), and likely the dose used. For example, Li et al. (2021) reported a quadratic response in milk yield with increasing live yeast doses ranging from 2 to 6 × 10^10^ CFU/d. In summary, the effect of live yeast in vitro is quite consistent, but the doses used in vitro are much greater than those used in vivo. Taking these previous studies together, there seems to be a need to define the exact scenarios (i.e., type of ration, site of action, and supplemental dose) where live yeast supplementation could be desirable.

### Bacteria-based DFM

In ruminants, most bacterial DFM have targeted intestinal health of young animals, mainly calves. Most milk replacers have some strains of *Enterococcus* spp. or *Lactobacillus* spp., which preferentially ferment nutrients into lactate inducing a drop in the pH (Kung et al., 2003; Riddel et al., 2010; Muck et al., 2018) that ultimately impacts the growth and survival of potentially harmful bacteria. A DFM based on *L. acidophilus* and *P. freudenreichii* was reported to improve feed efficiency in dairy cows in early, but not in mid-lactation cows (Boga and Gorgulu, 2007; Ferraretto and Shaver, 2015) due to a slight reduction in DMI with changes in milk yield. Philippeau et al. (2017) reported an increase in rumen pH when supplementing *Propionibacterium* spp. and *Lactobacillus* spp. to dairy cows. In adult cattle, the main bacterial DFM studied has been *M. elsdenii*, with most of the studies conducted in beef animals (Klieve et al., 2003; Henning et al., 2010). In dairy cattle,  Aikman et al. (2011) reported a positive response in ruminal pH when supplementing cows with 5 × 10^9^ CFU of *M. elsdenii*/d. Zebeli et al. (2012), however, reported no changes in ruminal pH when supplementing cows with 3.5 × 10^9^ CFU of *M. elsdenii*. In terms of cattle performance, results are variable. Hagg et al. (2010) found no changes in DMI or milk yield in dairy cows supplemented with 2.5 × 10^11^ CFU of *M. elsdenii*/d administered 3 times every 10 d. Stevens et al. (2017) evaluated the effect of supplementing pre-, post-, and pre- and postpartum cows with 2 × 10^10^ CFU of *M. elsdenii* and found an increase in milk yield only in third lactation cows, regardless whether supplementation was performed pre- or postpartum, which would indicate that a single dose would be sufficient to elicit a change. The reason why no response was observed in younger cows (first and second lactation) could be associated with their smaller BW and DMI, which limits ruminal supply of starch and rapidly fermentable carbohydrates that can cause acidosis. However, this is just a speculation as DMI was not measured in Stevens et al. (2017). Nevertheless, results from Aikman et al (2009) support this rationale as supplementing 5 × 10^9^ CFU of *M. elsdenii* resulted in increases in DMI and milk yield only in high- but not in low-producing dairy cows. Hence, inconsistency in the response could also be linked to differences in rations, doses of DFM used, and level of feed intake.

Another recent attempt to evaluate the effect of DFM in ruminants has consisted of a mixture of native microbes (*B. licheniformis* and *B. subtilis*) isolated from the rumen of cows classified as efficient at converting feed into milk. This combination of bacteria has been reported to improve milk performance and feed efficiency when supplemented at the rate of 9.6 × 10^9^ CFU/d in dairy cattle (Cappellozza et al., 2024). However, in a similar study, where the same combination of DFM was supplemented at 6 × 10^11^ CFU/d did not result in milk performance or feed efficiency of rumen-fistulated Holstein cows (Lamontagne et al., 2023). More recently, Terré et al. (2024) reported improvements in feed efficiency of early- to mid-lactating dairy cows offered a 28.5% starch diet with the addition of 9.6 × 10^9^ CFU/d of *B. licheniformis* and *B. subtilis*. Peng et al. (2011) reported an improvement in milk production of dairy cows without affecting DMI and thus increasing feed efficiency when supplementing with 5 × 10^10^ or 1 × 10^11^ CFU/d of *B. subtilis natto* only. Some combinations of naturally occurring microorganisms isolated from high-efficient cows has been evaluated for their potential to improve feed efficiency and performance. Valldecabres et al. (2022) reported improvements in milk yield when supplementing cows with a mixture of *Clostridium beijerinckii*, *Pichia kudriavzevii*, *Ruminococcus bovis*, and *Butyrivibrio fibrisolven*s, but the response was not observed when the two last microorganisms were removed from the mixture. A follow-up study (Marinho et al., 2024) found similar results with further improvements in milk production and feed efficiency when supplementing the combination of four microorganisms.

In summary, performance response due to supplementation of bacteria-based DFM seems to be more consistent than those obtained with yeast-based DFM. Nevertheless, as it seems to be the case for yeast, the dose at which bacteria-based DFM are supplemented may also play a role behind the discrepancy in the results, as responses in milk yield in dairy cows were shown to be dose-dependent when supplementing *Bacillus*-based DFM (Choonkham et al., 2020).

### Future opportunities

Most DFM-related research in adult dairy cattle has targeted improvements in rumen fermentation, and the potential benefits of using DFM targeting both the small and large intestines may represent an opportunity. To the best of our knowledge, only two studies have evaluated the potential effects of DFM on the intestine of ruminants, with severe limitations. Using three cows per treatment, Wu et al. (2024) tested a combination of encapsulated *B. coagulans* and *S. boulardii* and detected a positive effect on milk yield. The study by Chida et al. (2021) assessed the impact of supplementing *L. plantarum* in five transition dairy cows, but the presence of the DFM was confounded with energy level of the diet and it is difficult to extract conclusions. A potential DFM with demonstrated activity in the intestine of cattle is *Bacillus* spp. which may support the function and integrity of the lower gastrointestinal tract through different modes of action (Sun et al., 2010; Copani et al., 2020; Santano et al., 2020; Segura et al., 2020; Luise et al., 2022; Boll et al., 2024b). Supplementing *B. subtilis* spores at the rate of 3 × 10^9^ CFU/d for 16 wk in dairy cows increased milk yield and milk protein yield with no changes in feed digestibility (Souza et al., 2017).

Maintaining gut length and absorptive surface requires energy and amino acids, and to ensure that the intestinal lengthening pays off, the energy needed to maintain the increased intestinal surface must be justified by an increase in energy balance elicited by the lengthening of the intestine (Chevalier et al., 2015). Mammals exposed to cold weather, including cattle, consume more feed to compensate for the additional energy needed to maintain body temperature. In mice, it has been shown that the increase in intake due to cold exposure affects the intestinal microbiome in a way that facilitates nutrient absorption in the intestine. Chevalier et al. (2015) showed that intestinal absorption capacity was increased when cold-exposed mice increased DMI and consequently the relative abundance of *Akkermansia muciniphila* decreased. This reduction elicited an increase in intestinal mucin (as *A. muciniphila* is a mucin-degrading microorganism) and in turn that resulted in an increased absorption capacity of intestinal glucose. The authors also demonstrated that mice exposed to cold and inoculated with *A. muciniphila* lost weight, whereas their counterparts, not supplemented with *A. muciniphila*, were able to sustain growth rates >1 g/d. Another study demonstrating the potential of modifying the intestinal microbiome was conducted in mice using fecal microbiome from bears. Sommer et al. (2016) characterized the fecal microbiome of Brown bears in summer and in winter and found substantial differences in Bacillota (greater in summer vs. winter) and Bacteroidetes (greater in winter vs. summer). Then, gnotobiotic mice receiving the same diet were inoculated with bear fecal matter obtained in summer or winter. Mice that were inoculated with the summer microbiome grew about 30% more and deposited nearly double the amount of fat than mice inoculated with the winter microbiome. When mice were exposed to an oral glucose tolerance test, those inoculated with the summer flora maintained lower blood glucose than those inoculated with winter fecal matter. Lastly, by targeting changes in the intestinal microbiome, there is a possibility of inducing epigenetic changes at the host level that may help the animal to adapt to a given situation such as dietary or environmental change. This type of epigenetic mechanism has already been described in fish (Hansen et al., 2023).

## Summary and Conclusions

The livestock industry is continuously evolving to integrate sustainable and innovative technologies to enhance soil and plant stability, preserve feed quality, and optimize animal health and productivity. The growing interest in microbial solutions to improve production efficiency in beef and dairy systems was the focal point of the Ruminant Nutrition Symposium “Novel microbial solutions to optimize production efficiency in beef and dairy systems” held during the 2024 ASAS Annual Meeting. This manuscript reviewed the presented research on bacterial-based microbial solutions, particularly their mechanisms of action in silage preservation, as well as performance and health of beef and dairy cattle.

Microbial solutions play a crucial role in silage preservation, particularly through bacterial inoculants that improve fermentation processes and aerobic stability. The combination of homofermentative and heterofermentative bacteria enhances the nutritional quality of silage, mitigating undesirable fermentation patterns while improving digestibility and feed efficiency. The research highlighted the benefits of such microbial solutions in reducing DM losses and increasing nutrient availability for cattle. The integration of microbial solutions in cattle diets has also demonstrated promising benefits in modulating gut health, nutrient metabolism, and immune function. *Bacillus*-based DFM contributes to improved rumen fermentation, increased nutrient absorption, and enhanced host immunity. The application of *Bacillus*-based DFM in cow-calf, backgrounding, feedlot, and dairy systems has shown potential for improving feed intake and digestion, performance traits, and overall productivity.

Despite the advancements in microbial solutions including *Bacillus*-based DFM, further research is needed to optimize bacterial strains, dosages, and combinations tailored to specific production systems and environmental conditions. As beef and dairy industries continue to pursue sustainable alternatives to chemical and antimicrobial interventions, microbial solutions emerge as a viable strategy to enhance productivity while ensuring environmental stewardship. The continued investigation and application of these technologies will play a vital role in shaping the future of silage production and cattle nutrition.

## Abbreviations

BW: body weight
BCS: body condition score
CP: crude protein
DFM: direct-fed microbials
DM: dry matter
DMI: dry matter intake
FCM: fat-corrected milk
GRAS: **“**Generally Recognized As Safe”
LAPB: lactic acid-producing bacteria
LAUB: lactic acid-utilizing bacteria
NDF: neutral detergent fiber
NH_3_-N: ammonia-N
TMR: total mixed ration
VFA: volatile fatty acid
